# 2438: Dose-dependent nature of cocaine infusions on cardiovascular hemodynamics

**DOI:** 10.1017/cts.2017.138

**Published:** 2018-05-10

**Authors:** Salvatore Carbone, Benjamin Van Tassell, Antonio Abbate, Justin Canada, Leo F. Buckley, Sade Johns, Dinesh Kadariya, F. Gerard Moeller

**Affiliations:** Virginia Commonwealth University, Richmond, VA, USA

## Abstract

OBJECTIVES/SPECIFIC AIMS: Cocaine use is a significant health problem in the United States and associated with increased risk of adverse cardiovascular outcomes. Our goal was to evaluate the effects of rapid cocaine infusions on cardiovascular hemodynamics among patients with cocaine abuse disorder. METHODS/STUDY POPULATION: Patients with a history of cocaine abuse but no overt cardiovascular disease received 4 consecutive intravenous infusions of cocaine (0, 10, 20, 40 mg) given in randomized, double-blinded order. The infusion procedure was repeated on 2 consecutive days (4 infusions each day). Following each dose, patients underwent continuous monitoring via fingertip plethysmography for 30 minutes, followed by an additional 30 minutes washout procedure. Patients were surveyed throughout this timeline to record symptoms of cocaine response. Finger tracings were then used to calculate arterial pressure curves and parameters of heart rate, blood pressure, cardiac output, stroke volume, and systemic vascular resistance according to device-specific algorithms. Mean values were calculated over the entire 30 minutes follow-up and peak values were defined as the maximum value sustained over any 60-second interval during the follow-up period. RESULTS/ANTICIPATED RESULTS: Seven patients were enrolled and received cocaine infusions of 2 consecutive days. Cocaine dose was positively associated with mean cardiac output (*R*=0.489, *p*<0.001), peak diastolic blood pressure (*R*=0.435, *p*=0.001), mean heart rate (*R*=0.401, *p*=0.003), peak systolic blood pressure (*R*=0.399, *p*=0.003), peak mean arterial pressure (*R*=0.362, *p*=0.008), mean systolic blood pressure (*R*=0.399, *p*=0.003), +dP/dt (*R*=0.346, *p*=0.012), and peak heart rate (*R*=0.334, *p*=0.015). Hemodynamic parameters were also predictive of patient-reported symptoms of cocaine response. DISCUSSION/SIGNIFICANCE OF IMPACT: These data confirm the known pharmacologic effect of cocaine to prevent reuptake of neurotransmitters and demonstrate the feasibility of conducting a noninvasive assessment of cardiovascular hemodynamics as a measure of responsiveness to cocaine infusions. This procedure also provides a benchmark to evaluate the potential impact of pharmacologic treatments on cocaine-induced hemodynamic changes and patient perceptions of cocaine response.

